# Binge Drinking among adolescents is related to the development of Alcohol Use Disorders: results from a Cross-Sectional Study

**DOI:** 10.1038/s41598-018-29311-y

**Published:** 2018-08-22

**Authors:** Giovanni Addolorato, Gabriele A. Vassallo, Giulio Antonelli, Mariangela Antonelli, Claudia Tarli, Antonio Mirijello, Adwoa Agyei-Nkansah, Maria C. Mentella, Daniele Ferrarese, Vincenzina Mora, Marco Barbàra, Marcello Maida, Calogero Cammà, Antonio Gasbarrini, Alcohol Related Disease Consortium*, Giovanni Bruno, Giovanna D’Angelo, Fabio Del Zompo, Teresa Di Rienzo, Daniela Feliciani, Fabrizio Forte, Vanessa Isoppo, Lucrezia Laterza, Francesca Mangiola, Carolina Mosoni, Margherita Rando, Luisa Sestito

**Affiliations:** 1 Department of Internal Medicine and Gastroenterolog, Fondazione Policlinico Universitario A. Gemelli IRCCS, Roma – Università Cattolica del Sacro Cuore, Rome, Italy; 2 ”Alcohol Related Diseases” Unit, Department of Internal Medicine and Gastroenterology, Fondazione Policlinico Universitario A. Gemelli IRCCS, Roma – Università Cattolica del Sacro Cuore, Rome, Italy; 3 Department of Surgical and Medical Sciences and Translational Medicine, Sapienza University of Rome, Rome, Italy; 4 Department of Medical Sciences, IRCCS Casa Sollievo della Sofferenza Hospital, San Giovanni Rotondo, Italy; 5 Department of Medicine, University of Ghana School of Medicine and Dentistry, Accra, Ghana; 6 Biomedical Department of Internal and Specialized Medicine, University of Palermo, Palermo, Italy

## Abstract

Binge drinking (BD) is a common pattern of alcohol consumption among adolescents. At present few data are available on the possible relationship between BD and alcohol use disorders (AUD) in adolescents. The aim of this study was to assess the prevalence of BD and relationship between BD behavior and AUD among adolescents. A total of 2704 students attending 10 purposively selected high schools from three Italian provinces were surveyed. Questionnaires regarding socio-demographic data, pattern and amount of alcohol intake, smoking habits, use of illicit drugs, and physical activity were administered. AUD and affective disorders were also evaluated. Alcohol intake was reported by 2126 participants; 1278 reported at least one episode BD in the last year and 715 in the last month. A diagnosis of AUD was made in 165 adolescents. The prevalence of AUD was higher in adolescents that reported BD behavior than in those that did not report BD (11.6% vs 0.9%, respectively; p < 0.0001). Logistic regression showed a positive relationship between a diagnosis of AUD and BD behavior (OR 9.6; 95% CI 4.7–22·9; p < 0.0001). In conclusion alcohol consumption with the pattern of BD among adolescents is highly related to development of AUD.

## Introduction

Alcohol use disorders (AUD) are chronic and relapsing conditions characterized by harmful alcohol intake, compulsive drinking, psychological and physical dependence. AUD are associated with substantial disability, reduced quality of life, accidents and violence^1^.

AUD are responsible for over 2.5 million deaths every year worldwide^2^. According to World Health Organization (WHO) the worldwide prevalence of AUD is 4.1%, although industrialized countries show higher prevalence^3^.

According to the National Survey on Drug Use and Health, AUD prevalence is dramatically increasing among adolescents^3^. The diagnosis of AUD in adolescent is difficult because the available diagnostic criteria have significant limitations when applied to this population^4^. The peak of AUD in adolescent is observed at the age of eighteen, although different studies showed that fourteen is the threshold age from which the frequency of AUD increase^5^.

At present, in the US, 90% of alcohol consumed by teenagers is in the form of binge drinking (BD)^3^. BD is defined as the consumption of large amounts of alcoholic beverages in a single setting^6^. The cut-off for BD has typically been set at 5 or more drinks in a single occasion, because this amount quickly takes blood alcohol levels to 80 mg/dL^7^. However, this cut-off has been defined for adults. Given youths’ lower weight the cut-off could be lower^8^. BD habit during adolescence is associated with poorer performance in neurocognitive tests, particularly in executive functions and verbal memory, and with a greater frequency of brain structural alterations such as significant decrease in the gray matter of the dorsomedial prefrontal cortex on magnetic resonance imaging^9^. Associated with BD among adolescents are greater number of health risk behaviors, like smoking, illicit drug use, unprotected sexual activities, violence and drunk driving^8,10^.

Smoking is also considered a major cause of morbidity and mortality^11^, and it is strongly associated with alcohol consumption, especially in young people. Tobacco and alcohol act synergistically, increasing the risk for liver, cardiovascular and neoplastic diseases; especially if this habit is developed during adolescence^12^.

Illicit drug use often begins during adolescence. These behaviors are linked to increased morbidity and mortality and represent major public health challenges^11^. Co-administration of alcohol and illicit drugs increase the risk of organ damage particularly in adolescents^13^.

Adolescence is characterized by the rapid development of brain systems mediating reward and by changes in the secretion of stress-related hormones, events that might participate in the initiation pattern of alcohol and drug consumption. Experimental evidence suggests that early exposure to alcohol sensitizes the neuro-circuitry of addiction and affects chromatin remodeling. These events could induce abnormal plasticity in reward-related learning processes that contribute to adolescents’ vulnerability to drug addiction^14^. Moreover, repeated high-doses of alcohol intake followed by periods of total abstinence can sensitize γ-aminobutyric acid and N-methyl-d-aspartate receptors in the central nervous system, that control excitability, neural plasticity and memory^15^. This receptorial sensitization, in particular during adolescence, may induce withdrawal symptoms that could perpetuate alcohol intake behavior leading to AUD. High extracellular dopamine gradients have been described in adolescent rats exposed to BD^16^. This mechanism may be involved in the reinforcing effects of alcohol that could increase in adolescents with binge drinking behavior the risk to develop AUD. Starting from these neurobiological evidences we hypothesized that BD pattern during adolescence could represent a risk factor for the development of AUD and other health risk behaviors (smoking and use of illicit drugs). However, at present there are few information on the possible relationship between BD behavior and AUD or drug use in adolescents. In particular, an Australian study investigated a possible association between AUD and BD and their temporal separation. This study showed an increased risk for AUD in the early 20 s (21–24 y.o.) among BD teens (14–15 y.o.)^17^. Similar conclusions have been shown by a study conducted among native Mexican americans^18^.

An Italian study by Martinotti and colleagues showed a high and alarming prevalence of BD among Italian adolescents^19^. As suggested by some investigators, these aspects could be related to difficulties in family functioning^20^.

The present cross-sectional study was conducted among Italian adolescents attending high school, with the primary aim to assess the prevalence of BD and AUD among adolescents. These findings are presented in accordance with the Strengthening the Reporting of Observational Studies in Epidemiology (STROBE) statement^21^.

## Results

Among the 2704 participants enrolled, 1605 (59%) were females and 1077 were males (40%). Complete socio-demographic data and anthropometric characteristics are reported in Table 1.

**Table 1:** Sociodemographic characteristics of the 2704 subjects enrolled in the study.

Male, *n* (%)	1077(40)
Female, *n* (%)	1605(59)
Subjects with missing data about gender, *n*	22
Age, mean +/− SD; range	16.2 +/− 1,5; 13–20
Adjusted BMI categories for children, n(%)	
Thinness	57(2)
Normal weight	2232(84)
Overweight	305(12)
Obese	52(2)
Subjects with divorced parents, n (%)	383(14)
Subjects with father’s education >8 years, *n* (%)	2184(81)
Subjects with mother’s education >8 years, *n* (%)	2294(85)
Subjects that live in family with more than 3 family members, *n* (%)	2010(74)
Subjects with more than one sibling, *n* (%)	622(23)
Subjects attending high school in Rome, *n* (%)	1117(41)
Subjects attending high school in Latina, *n* (%)	1108(41)
Subjects attending high school in Frosinone, *n* (%)	479(18)
Subjects who practice weekly physical activity, *n* (%)	1923(71)
Subjects who practice competitive physical activity, *n* (%)	725(27)

A total of 2126 (79%; 95% confidence interval (CI) 78–81%) adolescents reported alcohol consumption, out of which 1473 (55%; 95% CI 53–57%) reported occasional alcohol intake, 597 (22%; 95% CI 20–24%) at least once a week and 33 (1.6%; 95% CI 0.0–3.6%) every day. A total of 270 (11%; 95% CI 9–13%) adolescents consumed aperitifs and digestives, 404 (16%; 95% CI 14–19%) spirits, 961 (39%; 95% CI 37–41%) beer and 247 (10%; 95% CI 8–12%) wine. A total of 1185 (44%; 95% CI 42–46%) consumed alcohol out of meals. Complete data about amount and pattern of alcohol intake are reported in Table 2. For those who report alcohol use, 1278 (48%; 95% CI 46–50%) had at least one episode of BD in the last year and 715 (27%; 95% CI 25–29%) in the last month.

**Table 2:** Data about alcohol consumption of the 2704 subjects enrolled in the study*.

Subjects that report alcohol intake, *n* (%; CI)	2126 (79; 78–81)
Subjects that occasionally drink, *n* (%; CI)	1473 (55; 53–57)
Subjects that drink weekly, *n* (%; CI)	597 (22; 20–24)
Subjects that drink every day, *n* (%; CI)	33 (1.2; 0.0–3.6)
Subjects with missing data about frequency of drinking, *n*	23
Subjects that consume aperitifs and digestives, *n* (%; CI)	270 (11; 9–13)
Subjects that drink beer, *n* (%; CI)	961 (39; 37–41)
Subjects that consume spirits, *n* (%; CI)	404 (16; 14–19)
Subjects that drink wine, *n* (%; CI)	247 (10; 8–12)
Subjects with missing data about type of alcoholic beverages consumed, *n*	244
Subjects that drink alcohol out of meals, *n (*%; CI)	1185 (44; 42–46)
Subjects that occasionally drink alcohol out of meals, *n (*%; CI)	764 (29; 27–31)
Subjects that weekly drink alcohol out of meals, *n*(%; CI)	346 (13; 11–15)
Subjects that every day drink alcohol out of meals, *n*(%; CI)	15 (0.57; 0.00–2.53)
Subjects with missing data about frequency of drinking alcohol out of meals, *n*	60
Subjects that drink with the pattern of binge drinking in the last year, *n* (%; CI)	1278 (48; 46–50)
Subjects that drink with the pattern of binge drinking in the last month, *n* (%; CI)	715 (27; 25–29)

Among adolescents that reported alcohol consumption, 1956 (72%; 95% CI 70–74%) declared that neither parents, relatives, friends, general practitioner or other health operators have educated them on the risk of drinking or advised them to stop drinking.

According to the Alcohol Use Disorders Inventory Test (AUDIT) criteria, a diagnosis of AUD was made in 165 (6.1%; 95% CI 5.3–7.1%) of the participants; in particular harmful drinking behavior was diagnosed in 132 (4.9%; 95% CI 4.1–5.8%) and alcohol dependence in 33 (1.2%; 95% CI 0.4–2.1%) adolescents.

**Figure 1:**
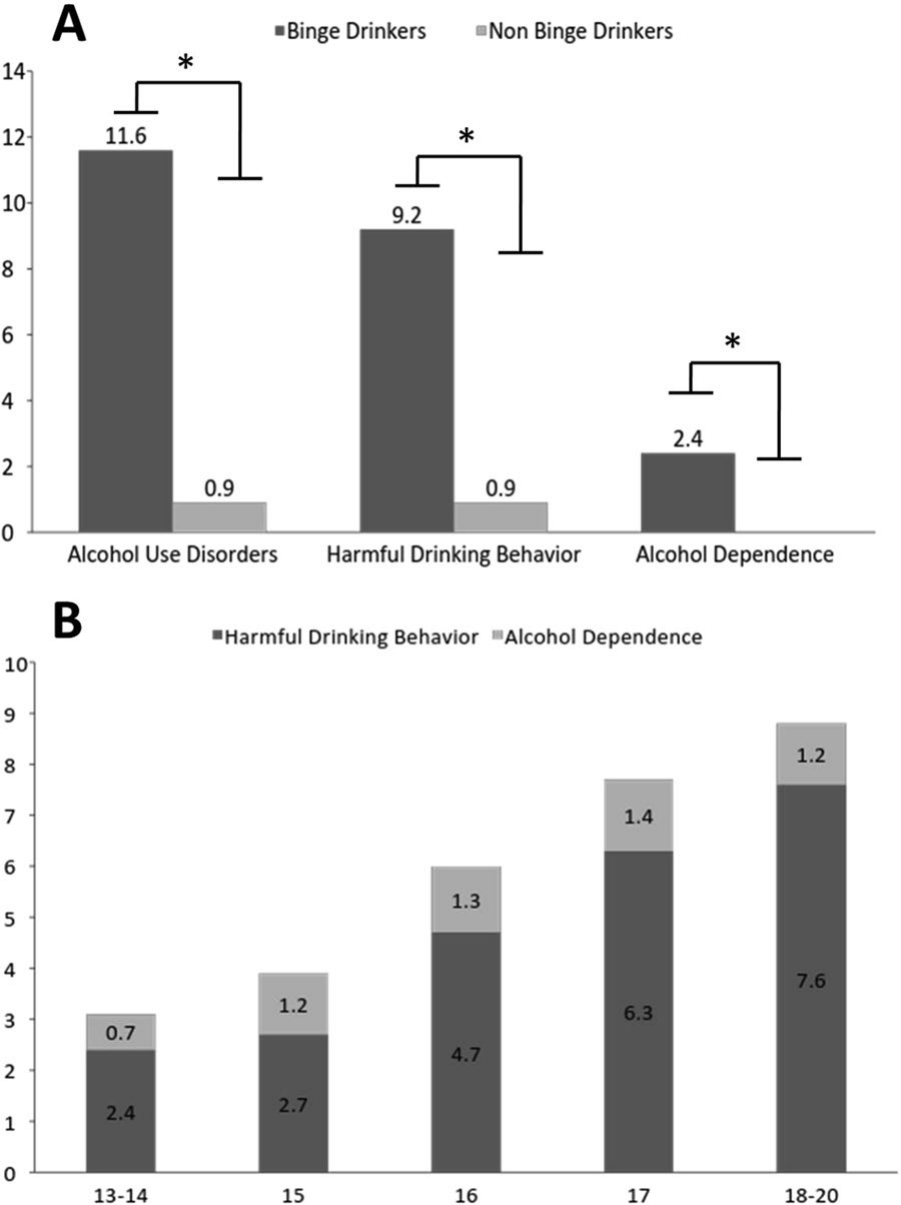
(**A**) Prevalence of participants with diagnosis of Alcohol Use Disorder, Harmful Drinking Behaviour and Alcohol Dependence among Binge Drinkers and not Binge Drinkers. (**B**) Prevalence of participants with diagnosis of Alcohol Use Disorder based on age.

The prevalence of AUD was higher among adolescents that reported BD (12%; 95% CI 10–13%) than in those that did not (0.87%; 95% CI 0.42–1.79%) (OR 12; 95% CI 6–26; p < 0.0001) (Fig. 1). In particular 9.2% (95% CI 7.6–10.9%) of participants that reported BD had a diagnosis of harmful drinking behavior compared to 0.87% (95% CI 0.37–1.48%) of those who did not binge (OR 11; 95% CI 5–23; p < 0.0001). Moreover, a diagnosis of alcohol dependence was made in 2.4% (95% CI 0.7–4.0%) of the adolescents drinking with the pattern of BD whereas none with alcohol dependence was found in the group that was not binge drinking (Fig. 1). The prevalence of AUD increased with age, from 3.1% (95% CI 1.7–5.3%) among those aged 13–14 to 8.8% (95% CI 6.7–11.4) among those aged 18–20 (Fig. 1).

Among all participants, 787 (29%; 95% CI 28–31%) smoked cigarettes. Specifically, 201 (7.5%; 95% CI 5.8–9.2%) smoked cigarettes occasionally, 154 (5.8%; 95% CI 4.1–7.5%) at least once a week and 422 (16%; 95% CI 14–17%) every day. Complete data about smoking habits are reported in Table 3.

**Table 3:** Data about smoking habit and use of illicit drugs of the 2704 subjects enrolled in the study*.

Subjects with smoking habit, *n* (%; CI)	787 (29; 28–31)
Subjects that occasionally smoke, *n* (%; CI)	201 (7.5; 5.8–9.2)
Subjects that weekly smoke, *n* (%; CI)	154 (5.8; 4.1–7.5)
Subjects that every day smoke, *n* (%; CI)	422 (16; 14–17)
Subjects with missing data about frequency of smoke, *n*	10
Subjects that smoke less than 5 cigarettes at day, *n* (%; CI)	432 (16; 14–18)
Subjects that smoke between 5 and 10 cigarettes at day, *n* (%; CI)	199 (7.5; 5.8–9.2)
Subjects that smoke between10and 15 cigarettes at day, *n* (%; CI)	72 (2.7; 1.1–4.4)
Subjects that smoke between 15 and 20 cigarettes at day, *n* (%; CI)	25 (0.94; 0.00–2.65)
Subjects that smoke more than 20 cigarettes at day, *n* (%; CI)	11 (0.41; 0.00–2.12)
Subjects with missing data about the amount of cigarettes smoked, *n*	48
Subjects that use illicit drugs, *n* (%; CI)	446 (18; 16–19)
Subjects that use Cannabis, *n* (%; CI)	417 (17; 15–18)
Subjects that use Cocaine, *n* (%; CI)	16 (0.59; 0.36–0.96)
Subjects that use Heroin, *n* (%; CI)	9 (0.33; 0.18–0.63)
Subjects that use Amphetamine (Ecstasy or similar), *n* (%; CI)	19 (0.70; 0.45–1.09)
Subjects with missing data about type of illicit drug used, *n*	29
Subjects that occasionally use illicit drug, *n* (%; CI)	288 (11; 10–13)
Subjects that weekly use illicit drug, *n* (%; CI)	98 (3.6; 2.3–4.9)
Subjects that every day use illicit drug, *n* (%; CI)	48 (1.8; 0.5–3.1)
Subjects with missing data about frequency of use illicit drug, *n*	12

Among all participants, 446 (18%; 95% CI 16–19%) reported use of illicit drugs. Among those reporting use of illicit drugs, 417 (17%; 95% CI 15–18%) reported cannabis use, 16 (0,59%; 95% CI 0.36–0.96%) cocaine, 9 (0,33%; 95% CI 0.18–0.63%) heroine and 19 (0.70%; 95% CI 0.45–1.09%) amphetamine. Someone reported multiple drug use. Among illicit drugs users, 288 (11%; 95% CI 10–13%) used them occasionally, 98 (3.6%; 95% CI 2.3–4.9%) at least once a week and 48 48 (1.8%; 95% CI 0.5–3.1%) every day. Complete data about use of illicit drug are reported in Table 3. Age-stratified data are reported in Table 4.

**Table 4:** Percentage and confidence interval stratified for age of subjects with alcohol intake, binge drinking behavior, diagnosis of Alcohol Use Disorder, smoking habit and use of illicit drugs.

	13–14	15	16	17	18	19–20
Subjects that report alcohol intake, % (CI)	58 (54–63)	70 (66–74)	80 (76–83)	86 (83–89)	93 (90–95)	91 (85–95)
Subjects that drink with the pattern of binge drinking, % (CI)	26 (22–30)	38 (34–42)	46 (42–50)	58 (53–62)	64 (59–68)	62 (54–69)
Subjects with a diagnosis of Alcohol Use Disorders, % (CI)	3 (2–6)	4 (3–6)	6 (4–8)	8 (6–11)	9 (6–12)	9 (5–14)
Subjects with smoking habit, % (CI)	18 (15–22)	25 (21–29)	24 (21–28)	37 (33–41)	38 (34–43)	39 (32–47)
Subjects that use illicit drugs, % (CI)	18 (15–22)	25 (21–29)	24 (21–28)	37 (33–41)	38 (34–43)	39 (32–47)

Among all participants, 1727 (65%; 95% CI 63–67%) showed STAI-Y1 score > 40, 1559 (59%; 95% CI 57–61%) a STAI-Y2 score > 40 and 334 (13%; 95% CI 11–14%) a ZUNG scale score > 50.

Logistic regression analyses showed a positive relationship between alcohol consumption and male gender (OR 1.9; 95% CI 1.4–2.4; p < 0.0001), age (OR 1.6; 95% CI 1.5–1.7; p < 0.0001), adolescents with divorced parents (OR 1.4; 95% CI 1.1–1.9; p = 0.017), smoking habits (OR 5.7; 95% CI 3.5–9.9; p < 0.0001), use of illicit drugs (OR 6.3; 95% CI 3.2–14.1; p < 0.0001) and STAI-Y1 score > 40 (OR 1.3; 95% CI 1.0–1.7; p = 0.0023; Nagelkerke’s pseudo R-squared 0.282) (Table 5).

**Table 5:** Odds ratios and corresponding 95% confidence intervals from logistic regression multivariable models for Alcohol consumption, Binge drinking behavior, and Alcohol Use disorders.

	N	Alcohol consumption^(a)^			Binge Drinking^(a)^			Alcohol Use Disorder^(a)^		
		%	OR (95% CI)	p	%	OR (95% CI)	p	%	OR (95% CI)	p
Total	2704	78.6			47.2			6.1		
Sex^(b)^										
Female	1605	75.2	1		43.4	1		6.2	1	
Male	1077	83.4	1.86 (1.47–2.37)	<0.001	52.6	1.45 (1.17–1.80)	<0.001	6.5	0.98 (0.66–1.45)	0.916
Age										
13–15	992	65.1	1		32.7	1		4.1	1	
16	577	80.0	1.96 (1.50–2.58)	<0.001	46.1	1.27 (0.96–1.67)	0.090	6.1	1.21 (0.76–1.94)	0.417
17	524	86.0	2.90 (2.11–4.04)	<0.001	57.4	1.77 (1.33–2.36)	<0.001	8.2	1.54 (0.98–2.41)	0.058
18–20	611	92.5	6.46 (4.47–9.62)	<0.001	63.1	1.98 (1.52–2.59)	<0.001	8.8	1.52 (1.00–2.34)	0.052
Parents’ marital status									—	—
Married parents	2321	77.2	1		45.8	1		5.9	—	—
Divorced parents	383	83.0	1.42 (1.07–1.90)	0.017	56.1	1.38 (1.01–1.90)	0.048	8.4	—	—
Smoking habit										
Non smokers	1917	72.0	1		35.5	1		3.0	1	
Smokers	787	94.7	5.73 (3.53–9.93)	<0.001	75.9	2.73 (2.16–3.47)	<0.001	14.7	1.73 (1.16–2.60)	0.008
Use of illicit drugs										
Non-users	2258	69.3	1		39.8	1		3.5	1	
Users	446	97.3	6.28 (3.23–14.14)	<0.001	85.0	3.20 (2.32–4.48)	<0.001	21.3	2.97 (2.01–4.42)	<0.001
STAI – Y1 score						—			—	—
≤40	977	72.4	1		44.8	—		5.0	—	—
>40	1727	79.7	1.31 (1.04–1.66)	0.023	48.6	—		6.4	—	—
STAI – Y2 score			—	—						
≤40	1145	73.9	—	—	44.9	1		4.9	1	
>40	1559	79.0	—	—	48.9	1.31 (1.06–1.63)	0.012	7.5	1.59 (1.14–2.25)	0.008
ZUNG SDS score			—	—		—				
≤50	2370	76.1	—	—	47.1	—		5.6	1	
>50	334	79.3	—	—	48.2	—		12.0	1.89 (1.18–3.02)	0.008
Binge drinking behaviour			—	—		—	—			
Non binge drinkers	1426	—	—	—	—	—	—	0.9	1	
Binge drinkers	1278	—	—	—	—	—	—	11.6	9.60 (4.70–22.90)	<0.001

^(a)^Variables selected by means of a stepwise regression procedure. ^(b)^The sum does not add up to the total because of some missing values.

Logistic regression analyses also showed positive relationship between BD and male gender (OR 1.4; 95% CI 1.1–1.7; p 0.002), age (OR 1.2; 95% CI 1.1–1.3; p < 0.0001), adolescents with divorced parents (OR 1.4; 95% CI 1.1–1.8; p 0.0066), smoking habits (OR 2.9; 95% CI 2.3–3.6; p < 0.0001), consumption of illicit drugs (OR 3.3; 95% CI 2.4–4.6; p < 0.0001) and STAI-Y2 score > 40 (OR 1.2; 95% CI 1.0–1.5; p = 0.0314; Nagelkerke’s pseudo R-squared 0.293) (Table 5).

Finally, there was a positive relationship between an AUDIT score > 8 and age (OR 1.1; 95% CI 1.0–1.3; p = 0.0142), smoking habits (OR 1.8; 95% CI 1.2–2.7; p = 0.0039), consumption of illicit drugs (OR 3.0; 95% CI 2.0–4.3; p < 0.0001), STAI-Y2 score > 40 (OR 1.6; 95% CI 1.2–2.3; p = 0.0059), ZUNGS score (OR 2.1; 95% CI 1.4–3.2; p = 0.0004), and Binge Drinking Behavior (OR 9.6; 95% CI 4.7–22.9; p < 0.0001; Nagelkerke’s pseudo R-squared 0.313) (Table 5).

## Discussion

This epidemiological survey on Italian adolescents has shown several disturbing trends. The above data reveal high rates of alcohol consumption among adolescents; moreover, many of them engage in drinking practices such as binge drinking and daily drinking. Given that adolescents are more susceptible to alcohol-related injury than adults because their liver enzymes are not completely expressed to adequately metabolize alcohol^22^, total alcohol abstinence during adolescence should be mandatory. However, despite the high percentage of drinking adolescents found in the present study, nobody, including parents and health operators, had ever tried to explain to them the detrimental effects of drinking or advised them to refrain from drinking. Basing on these observations, programs enhancing the consciousness about alcohol related risks (i.e. school teaching programs, media etc.) should be warranted.

Among students who consume alcohol, 47% reported BD in the last year. These figures on the high prevalence of BD are in line with previous international^6,8,10^ and national studies^19,20^. BD is a very harmful behavior associated with complications such as alcohol intoxication (blackouts, hangovers, etc.), negative behavioral effects (violence, unsafe sexual intercourse), deterioration of work performance, use of illicit drugs and premature death (particularly due to car crashes and/or suicides)^14^. In addition, BD leads to premature dysfunction of the brain, cardiovascular and gastrointestinal systems^12^.

According to AUDIT, our data shows that about 6% of interviewed participants meet the criteria for AUD. The prevalence of AUD in our cohort is higher than previously reported in the Italian population (1%) and higher than the worldwide prevalence in general population of 4.1% according to the World Health Organization^3^.

Previous studies show that the pattern of BD could be involved in AUD development during young adulthood^17^, although few data are at present available on this possible relationship. The present study shows a relationship between a diagnosis of AUD and BD behavior in adolescents. The prevalence of AUD was significantly higher in students with BD compared to those who did not binge on alcohol. The prevalence of harmful drinking behavior was about 10 times higher in binge-drinkers than in non-binge-drinkers; while AUDIT criteria for alcohol dependence were present only in students who reported BD. Thus, it is conceivable that BD represents in adolescents a risk factor for the development of AUD, including alcohol dependence, with possible onset of severe alcohol problems in adulthood. This hypothesis is supported by previous observational studies showing as BD typically emerges in adolescence anticipated the peak of AUD in young adulthood^17–20,23^.

A role of anxiety as factor able to increase the risk of alcohol dependence in actively drinking subjects have been reported^24^. Accordingly, in our sample, state and trait anxiety were associated with binge drinking behavior and with a diagnosis of AUD.

In addition to the high rates of alcohol use among this cohort, the data also demonstrated the common co-occurrence of additional use of tobacco, illicit drugs among binge users similar to the recent observations by Joffer *et al*.^25,26^. Moreover, previous studies showed that BD is associated with worse levels of tobacco dependence and poorer smoking treatment outcomes. Alcohol consumption and smoking habits are independent risk factors for cancer and cardiovascular disease and when used simultaneously they interact with each other synergistically elevating the risk of developing these diseases^27^.

Our data are also in line with previous studies showing that BD is a risk factor for illicit drug use^10,17^. The relationship between heavy drinking, smoking habits and use of illicit drugs might be related to a hedonistic youth culture. Moreover, BD might increase the individuals’ risk of making ill-considered decisions about the use of illicit drugs. Finally, co-administration of alcohol and illicit drugs increases the risk of organ damage, especially in young adolescents^28^.

The main limitation of this study was the cross-sectional design which prevent to draft definitive conclusion on a causal relationship between BD and AUD. A further limitation was using a self-reported approach. Although Self-report surveys are simple and inexpensive, this method of obtaining data has been questioned in adult samples because its results in underestimation of consumption, social desirability and recall biases. However, when used with adolescents, self-reports have been shown to be reliable and valid^29^. Furthermore, a selection bias cannot be excluded since our study included only high school attending adolescents, excluding those most at risk adolescents who do not attend school and thus missing the lower social class data.

## Conclusion

Alcohol consumption and abuse among students is very alarming. BD is becoming increasingly frequent among Italian adolescents, and it is related with the development of AUD, including alcohol dependence. BD is also related with smoking habits and use of illicit drugs.

Educational school programs teaching the risks related to alcohol and binge drinking (including development of AUD) are mandatory. Further studies as needed to understand better the relationship between BD and AUD are needed. Moreover is important implementing policies to prevent alcohol consumption and BD among adolescent

## Methods

A cross-sectional study was conducted among Italian adolescents, aged between 13 and 20 years, attending high school. This study was approved by the Ethics Committee of the Catholic University of Rome and it was performed in accordance with relevant guidelines and regulations. The study is registered with ClinicalTrials.gov, Number: NCT03062189.

Ten high schools from the 3 Italian provinces or Rome, Latina and Frosinone were purposively selected to form the sample intended to be representative of both urban and rural area of the Lazio region. In order to achieve this goal, five distinct subdivisions were identified in the urban area of Rome and other five in the provinces (respectively, 3 in the province of Latina and 2 in the province of Frosinone). For any given subdivision, an invitation was sent to the school with the highest number of students enrolled. All the invited schooled accepted to take part in the survey. Of the 2785 students attending these ten high schools, 2704 participated to the survey, which took place form January to June 2015.

The remaining 81 (3%) students, didn’t participate either because they did not consent or were absent the day the questionnaires were administered. Adolescents who agreed to participate in the study were consented by trained physicians and psychologists with expertise in AUD. These physicians also provided information about the questionnaire to students and answered all questions raised about the study. Absolute anonymity was guaranteed to every participant. Written informed consent have been obtained from enrolled participants (if they were aged 18 years or above) or from their parents when they are underage

Questionnaires regarding socio-demographic data, anthropometric characteristics, pattern and amount of alcohol intake, smoking habits, use of illicit drugs and physical activity were administered.

A drink was defined as amount of alcoholic beverages containing 12 g of absolute alcohol. BD was defined as an alcohol consumption of more than 5 drinks within a two-hour period. Regarding pattern of drinking the following data were collected: type, amount and frequency of alcohol consumed, alcohol consumption in relation to meals, and episodes of BD in the last month and year.

The Italian version of the Alcohol Use Disorders Inventory Test (AUDIT) questionnaire was used to screen alcohol use disorders in the participants. AUDIT is a ten-question test developed by World Health Organization in 1982 to screen risky drinking. It has a good sensitivity and specificity in alcohol related problems. AUDIT has been also validated across genders and in a wide range of racial/ethnic groups and it is an appropriate tool to screen adolescent and college students^30^. Scoring the AUDIT is based on a 0–4 point scale. There is a relationship between the AUDIT scores and alcohol related problems. A total score above 8 indicates a diagnosis of AUD, scores between 8 and 15 indicates harmful drinking behaviour, while scores above 15 indicates the presence of alcohol dependence^31^.

AUD may be associated with several psychological and affective disorders. Among them, anxiety and depression have been reported in AUD patients, although it is still controversial if these disorders may be a cause or rather a consequence of AUD^32^. In this study state anxiety and current depression were evaluated respectively with the State-Trait Anxiety Inventory (STAI) test and the Zung self-rating depression scale (Zung-SDS).

The State-Trait Anxiety Inventory (STAI) is a psychological tool of 40 self-report items to assesses existing anxiety and predisposition to anxious reaction as a personality characteristic. It is structured by two axes (y1 for state anxiety and y2 for trait anxiety), both consisting of 20 multiple-choice items; each item has a score from 1 to 4; the total score of y1 and y2 axes can range from 20 to 80^33^. This test was selected for its simplicity, validity and reliability. The adolescents evaluated were grouped as high-anxious and low-anxious and a median value of 40 was used to distinguish between the two groups. A higher STAI scores suggest higher levels of anxiety^33^.

The Zung-SDS is an effective instrument in screening for depression with a positive predictive value of a diagnosis of depression between 88.7% and 92.3%^34^. The Zung-SDS contains 20 multiple items with a score from 1 to 4 each; a total score above 50 indicates a condition of depression.

Regarding smoking, frequency and number of cigarettes sticks smoked per day were investigated as well as frequency and type of illicit drugs using a questionnaire.

### Outcome measure

Primary outcome measures were the prevalence and relationship between binge drinking and Alcohol Use Disorders among adolescents. We additionally estimated relationship with smoking, use of illicit drugs and affective disorders with binge drinking.

### Statistical analysis

The sample size needed for this study was calculated considering all 14 predictors and the 3 different outcomes. With a sought power of at least 0.90, sample size was calculated in 1766 subjects. It was decided however to complete the sampling of all the consenting students attending the chosen institutes even after the wanted number was achieved.

Continuous variables were summarized as mean ± standard deviation. Categorical variables were summarized by means of contingency tables of frequencies and proportions. Body Mass Index (BMI) data were adjusted for age and sex using the bmi-for-ages z-scores published by the World Health Organization.

Binomial confidence intervals for prevalence estimates were calculated for dichotomous variables and simultaneous Sison-Glaz confidence intervals were used for multinomial variables. No missing data imputation was performed and all prevalence estimates were based on actual responses. Separates linear logistic multiple regression models were used to evaluate relationships between alcohol consumption and binge-drinking variables with a set of candidate potential predictors. Variable selection was performed by means of a manual iterative stepwise regression procedure described as follows: initial model fitted with all variables (minimal residual deviance model); subsequent variable removal leading to the best improvement in terms of minimum Akaike Information Criterion^35^; re-evaluation of any previously removed variable, one at a time; end of process with a minimal AIC set of variables: age and gender were always retained in the final model in order to provide properly adjusted estimates; the amount of variance explained was evaluated by means of the Nagelkerke’s Pseudo R-square index. All the analyses were made using the R computing environment (R Core Team 2017)^36^.

### Ethical approval

This study was approved by Ethical Committee of Catholic University of the Sacred Heart, Rome, Italy. All authors confirm that the study was performed in accordance with relevant guidelines and regulations.

### Transparency

The lead and corresponding author (Giovanni Addolorato) affirm that the manuscript is an honest, accurate, and transparent account of the study being reported; that no important aspects of the study have been omitted; and that any discrepancies from the study as planned have been explained.
