# Author Correction: Binge Drinking among adolescents is related to the development of Alcohol Use Disorders: results from a Cross-Sectional Study

**DOI:** 10.1038/s41598-018-33705-3

**Published:** 2018-10-16

**Authors:** Giovanni Addolorato, Gabriele A. Vassallo, Giulio Antonelli, Mariangela Antonelli, Claudia Tarli, Antonio Mirijello, Adwoa Agyei-Nkansah, Maria C. Mentella, Daniele Ferrarese, Vincenzina Mora, Marco Barbàra, Marcello Maida, Calogero Cammà, Antonio Gasbarrini, Alcohol Related Disease Consortium, Giovanni Bruno, Giovanna D’Angelo, Fabio Del Zompo, Teresa Di Rienzo, Daniela Feliciani, Fabrizio Forte, Vanessa Isoppo, Lucrezia Laterza, Francesca Mangiola, Carolina Mosoni, Margherita Rando, Luisa Sestito

**Affiliations:** 1 Department of Internal Medicine and Gastroenterolog, Fondazione Policlinico Universitario A. Gemelli IRCCS, Roma – Università Cattolica del Sacro Cuore, Rome, Italy; 2 ”Alcohol Related Diseases” Unit, Department of Internal Medicine and Gastroenterology, Fondazione Policlinico Universitario A. Gemelli IRCCS, Roma – Università Cattolica del Sacro Cuore, Rome, Italy; 3 Department of Surgical and Medical Sciences and Translational Medicine, Sapienza University of Rome, Rome, Italy; 4 Department of Medical Sciences, IRCCS Casa Sollievo della Sofferenza Hospital, San Giovanni Rotondo, Italy; 5 Department of Medicine, University of Ghana School of Medicine and Dentistry, Accra, Ghana; 6 Biomedical Department of Internal and Specialized Medicine, University of Palermo, Palermo, Italy

Correction to: *Scientific Reports* 10.1038/s41598-018-29311-y, published online 22 August 2018

The original version of this Article contained a typographical error in the spelling of the author Marcello Maida, which was incorrectly given as Marcello F. Maida. This has now been corrected in the PDF and HTML versions of the Article.

